# The Potential Role of Subclinical *Bordetella pertussis* Infection in Epilepsy

**DOI:** 10.3389/fcimb.2019.00302

**Published:** 2019-08-28

**Authors:** Keith Rubin, Steven Glazer

**Affiliations:** ILiAD Biotechnologies, Weston, FL, United States

**Keywords:** *Bordetella pertussis*, epilepsy, seizure, *Bordetella pertussis* toxin, subclinical infection

The etiology of epilepsy remains unknown in 14–39% of cases across multiple continents (Banerjee et al., 2009). Given the increased risk for seizures and epilepsy in children after symptomatic *Bordetella pertussis* (BP) infection (Olsen et al., 2015), an association recognized for nearly a century (Eley, 1930), we briefly review the evidence and propose a role for subclinical BP colonizing infections in epilepsy.

Subclinical BP infections are vastly more prevalent than reported pertussis (Ward et al., 2005). In multiple countries with high BP vaccination rates, evidence of subclinical BP infection is demonstrated in 4.8–7.1% of asymptomatic individuals by nasal swab PCR (Klement et al., 2003; Zhang et al., 2014; Naeini et al., 2015), and in 6.6–14.1% by serology indicative of infection during the past year (de Melker et al., 2006; De Greeff et al., 2010; Palazzo et al., 2016). Based on serology, investigators in the United States (US) acellular BP vaccine trial estimated the number of undocumented BP infections at 1 to 10 million cases in the US annually from 1997 to 1999 (Ward et al., 2005), years when the CDC reported approximately 7,000 cases per year (http://www.cdc.gov/pertussis/surv-reporting/cases-by-year.html), a ratio of up to 1,400 subclinical BP infections for every reported pertussis case.

Multiple lines of evidence support the hypothesis that subclinical nasopharyngeal BP colonizing infections have unrecognized clinical consequences including epilepsy. *B. pertussis* secretes pertussis toxin, which compromises the blood-brain barrier in human brain endothelium models (Kugler et al., 2007), as seen in epilepsy (Oby and Janigro, 2006). Murine respiratory BP infection induces inflammatory cytokines in the brain (Loscher et al., 2000), and intracerebroventricular pertussis toxin lowers drug-induced seizure thresholds (Durcan and Morgan, 1991), though findings documented in mice should be interpreted with caution. At the neuronal level, mechanistic plausibility is supported in that pertussis toxin increases excitatory neuronal glutamate release (Cullen et al., 1994) and decreases Gi/o receptor-mediated neuroinhibitory GABA activity (Padgett and Slesinger, 2010), as well as GABA receptor binding (Moss and Vaughan, 1988). In summary, mechanisms by which pertussis infection may play a causal role in epilepsy include immunologic and inflammatory responses to pertussis infection, direct action of pertussis toxin on neurons, and a combination of these factors.

Clinical observation also supports the association between BP and epilepsy. In children < 2 years of age admitted to the hospital with pertussis, new seizures were reported in 2.3%, and encephalopathy in 0.5% of patients (Halperin et al., 1999). In BP-associated encephalopathy, elevated antibody titers to BP toxins have been demonstrated with 10-fold higher concentrations in CSF compared with serum, indicating entry of BP antigens to the CNS (Grant et al., 1998). In Denmark between 1978 and 2011, the incidence of epilepsy at 10 years of age was 1.7% for patients with a history of hospital-diagnosed pertussis, and 0.9% in a matched cohort [HR 1.7 (95% CI, 1.3–2.1)] (Olsen et al., 2015). Almost all of the increased epilepsy risk occurred in the first 1.5 years after clinical pertussis, and did not vary with age at pertussis diagnosis.

Investigating the hypothesis that subclinical BP colonizing infections are a cause of epilepsy could begin by screening patients presenting with an initial idiopathic seizure. Subjects and controls could be tested for serum BP antibody titers and nasopharyngeal BP by swab PCR. In future BP-seizure risk analyses, BP vaccination status should be accounted for to avoid the confounding effects of vaccine-induced BP immunoglobulins on estimates of BP exposure history. The particular form of BP vaccination is also important since the diphtheria, tetanus toxoid and whole-cell pertussis vaccine (DTP) has been associated with febrile seizures (but not epilepsy) (Barlow et al., 2001), while the combination acellular pertussis vaccine (DTaP) has not (Huang et al., 2010). Of note, some historically reported associations between pertussis vaccination and neurologic disorders may be due to early unmasking of genetically determined disease such as Dravet syndrome in those with sodium channel gene SCN1A mutations (McIntosh et al., 2010). Since these mutations may occur without a prior family history, referral for specialty testing should be considered to help identify all potential causes of new onset seizures.

As subclinical BP colonizing infections are prevalent in highly BP-vaccinated populations, and non-human primate studies demonstrate the failure of DTP and DTaP to prevent nasopharyngeal BP colonization (Warfel et al., 2014), evidence suggests that current pertussis vaccines do not prevent nasopharyngeal BP colonization. Since the number of subclinical BP infections may be more than 1,000 times greater than clinically reported cases as noted above, it would not be surprising to observe a minimal or even lack of epilepsy risk reduction following DTP and DTaP vaccination.

In light of the available evidence, we suggest that a causal role for subclinical BP colonizing infection in epilepsy is plausible and worthy of further investigation. Regression analysis of epilepsy risk, incorporating BP screening assays, medical history, and pertussis vaccination status would be a compelling first step in assessing the potential relationship between epilepsy and subclinical BP infection.

## Author Contributions

KR and SG contributed equally to the preparation of this manuscript.

### Conflict of Interest Statement

KR and SG are employed by and hold an equity interest in ILiAD Biotechnologies, which is developing a vaccine for the prevention of *Bordetella pertussis*. ILiAD Biotechnologies had no role in the study design, analysis, and development of this opinion submission.
